# CCT196969 effectively inhibits growth and survival of melanoma brain metastasis cells

**DOI:** 10.1371/journal.pone.0273711

**Published:** 2022-09-09

**Authors:** Agathe Reigstad, Christina Frantzen Herdlevær, Emma Rigg, Tuyen Hoang, Ole Vidhammer Bjørnstad, Synnøve Nymark Aasen, Jasmin Preis, Claude Haan, Terje Sundstrøm, Frits Thorsen

**Affiliations:** 1 Department of Biomedicine, University of Bergen, Bergen, Norway; 2 Faculty of Health and Social Sciences, Western Norway University of Applied Sciences, Bergen, Norway; 3 Department of Life Sciences and Medicine, University of Luxembourg, Belvaux, Luxembourg; 4 Department of Neurosurgery, Haukeland University Hospital, Bergen, Norway; 5 Department of Clinical Medicine, University of Bergen, Bergen, Norway; 6 Molecular Imaging Center, Department of Biomedicine, University of Bergen, Bergen, Norway; Rutgers University, UNITED STATES

## Abstract

Melanomas frequently metastasize to the brain. Despite recent progress in the treatment of melanoma brain metastasis, therapy resistance and relapse of disease remain unsolved challenges. CCT196969 is a SRC family kinase (SFK) and Raf proto-oncogene, serine/threonine kinase (RAF) inhibitor with documented effects in primary melanoma cell lines *in vitro* and *in vivo*. Using *in vitro* cell line assays, we studied the effects of CCT196969 in multiple melanoma brain metastasis cell lines. The drug effectively inhibited proliferation, migration, and survival in all examined cell lines, with viability IC_50_ doses in the range of 0.18–2.6 μM. Western blot analysis showed decreased expression of p-ERK, p-MEK, p-STAT3 and STAT3 upon CCT196969 treatment. Furthermore, CCT196969 inhibited viability in two *B-Raf Proto-Oncogene* (BRAF) inhibitor resistant metastatic melanoma cell lines. Further *in vivo* studies should be performed to determine the treatment potential of CCT196969 in patients with treatment-naïve and resistant melanoma brain metastasis.

## Introduction

The incidence of melanoma is rising faster than any other cancer [1], and 44–75% of patients with metastatic melanoma will develop brain metastases during the course of their disease [2–4]. Melanoma brain metastasis (MBM) is associated with a poor prognosis. If left untreated, the median patient survival is only a few months [5–7]. Aberrant activation of the mitogen-activated protein kinase (MAPK) signalling pathway represents a central step in melanoma development by providing increased proliferation and survival of melanoma cells [8]. Mutations are frequently seen in the *NRAS proto-oncogene* (*NRAS*; 28%) and *BRAF* (52%) genes [9], and 80% of *BRAF*-mutated melanomas display the *BRAF*^*V600E*^ mutation [10].

Treatment of metastatic melanoma has improved over the last decade due to the use of targeted inhibitors and immunotherapies. Although targeted therapies offer a promising avenue for treatment of patients with *BRAF*-mutated melanoma, most patients experience relapse of disease within a few months [11]. There are major challenges with intrinsic and acquired resistance to BRAF inhibitors [8, 11], and use of BRAF inhibitors may cause paradoxical activation of the MAPK pathway, leading to increased tumour growth [12, 13].

Mechanisms of resistance to BRAF inhibitors include MAPK-dependent mechanisms such as novel *NRAS* mutations or *BRAF* amplifications, and MAPK-independent mechanisms [14] such as upregulation of the epidermal growth factor receptor (EGFR)-SFK-signal transducer and activator of transcription 3 (STAT3) pathway and the phosphoinositide 3-kinase (PI3K) pathway [15, 16]. The STAT3 pathway is frequently upregulated in BRAF inhibitor resistant melanoma [15], which can drive invasion and metastasis [15, 17]. Furthermore, SFKs are known to promote a neoplastic and metastatic phenotype [18] and is commonly upregulated in BRAF inhibitor resistant cells [19, 20]. Patients often develop BRAF inhibitor resistance shortly after single drug treatment with BRAF inhibitors [21]. Therefore, the median survival is only modestly increased for patients with MBM upon treatment with MAPK inhibitors [22, 23]. Thus, there is an urgent need for novel therapeutic options through targeting multiple signalling pathways besides MAPK to improve patient outcome.

It has been shown that the pan-RAF and SFK inhibitor CCT196969 targets mutated BRAF^V600E^, C-Raf Proto-Oncogene (CRAF) and SFKs in primary melanoma cell lines [24]. Furthermore, the drug inhibited cell proliferation more effectively than the vemurafenib analogue PLX4720 and hampered growth of *RAS* mutant melanomas [24]. Here, we report for the first time that CCT196969 effectively inhibits cell growth of MBM cell lines *in vitro*.

## Materials and methods

### Cell lines and cell culture

The H1, H2, H3, H6 and H10 cell lines were established in our laboratory from patient biopsies of human MBM, as previously described [25]. Written, informed consent was obtained from all patients before collection of tumour material. The Regional Ethical Committee (REC) approved tissue collection, storage of tumour material, and generation and use of cell lines (REC Approvals 2013/720 and 2020/65185). The H1 and H2 cell lines harbour the *BRAF*^*V600E*^ mutation, while the H3 cell line is *BRAF*^*L577F*^ mutated [26]. In addition, H3 possesses *NRAS*^*Q61H*^ and *EGFR* mutations, while the H6 and H10 cell lines also harbour the *BRAF*^*V600E*^ mutation (unpublished data from our lab). The cells were cultured in Dulbecco’s Modified Eagle’s Medium (DMEM, Sigma-Aldrich Inc., St. Louis, MO, USA) supplemented with 10% heat-inactivated fetal calf serum (FCS) (Thermo Fischer Scientific, Waltham, MA, USA), 4 times the prescribed amount of non-essential amino acids, 2% L-Glutamine, 100 IU/mL penicillin and 100 μL/mL streptomycin (all reagents from BioWhittaker, Verviers, Belgium).

The *BRAF*^*V600E*^-mutated melanoma cell line Wm3248 was purchased from Rockland (Limerick, PA, USA). The cells were cultured in RPMI-1640 Glutamax™ (Thermo Fischer Scientific) supplemented with 10% heat-inactivated FCS (Thermo Fischer Scientific), 50 IU/mL penicillin and 50 μL/mL streptomycin (BioWhittaker).

All cell cultures were maintained in a standard tissue culture incubator at 37°C with 100% humidity and 5% CO_2_. Cells were subcultured to 70–90% confluency before passaging, and the growth medium was exchanged twice a week. Cells were tested and found mycoplasma free before use and authenticated using short tandem repeat profiling within 6 months of use.

### Drug

CCT196969 and vemurafenib were purchased from ChemieTek (Indianapolis, IN, USA). Both drugs were dissolved in dimethylsulfoxide (DMSO, Sigma-Aldrich Inc.) at a stock concentration of 50 mM and stored as aliquots at -20°C until use.

### Monolayer cell viability assay

Monolayer MTS assays were performed to study cell viability upon exposure to different concentrations of CCT196969. H1, H2, H3, H6, H10 and Wm3248 cells were seeded in 96-well plates (Nunc, Roskilde, Denmark) at a cell density of 5 x 10^3^ cells/well in 100 μL growth medium. After 24 h, 100 μL growth medium containing CCT196969 was applied to the wells in final concentrations of 0.0001, 0.001, 0.005, 0.01, 0.05, 0.1, 1, 10 and 50 μM for all cell lines except H2. For H2, 100 μL growth medium containing CCT196969 was applied to the wells in final concentrations of 0.001, 0.005, 0.01, 0.05, 0.1, 1, 5, 10 and 50 μM. After 72 h of incubation, morphology pictures were captured using a Nikon TE2000 inverted microscope (Nikon Instruments Inc., Melville, NY, USA). Floating cells were removed and 100 μL fresh DMEM was added prior to morphology imaging. 20 μL of MTS solution (CellTiter 96™ Aqueous One Solution Cell Proliferation Assay, Promega Corporation, Fitchburg, WI, USA) was then added to each well, and absorbance was measured at 490 nm 4 h later, using a Multiskan FC Microplate photometer (Thermo Fischer Scientific), equipped with SkanIt software. Cell viability curves were generated using Prism 8 Software (GraphPad Software Inc., San Diego, CA, USA), and IC_50_ doses were calculated; i.e., the drug concentrations at which 50% of the cell viability was inhibited. The experiments for the H1, H2, H3, H6 and H10 were done in triplicates. The Wm3248 experiment was performed in duplicate.

### Tumour sphere viability assay

To study the effects of treatment on 3-dimensional tumour growth, a tumour sphere viability assay was performed. A base agar was prepared by mixing 2.4% of Difco Noble Agar in purified water (Becton Dickinson and Company, Sparks, USA) with growth medium to a final concentration of 0.6%. The agar was kept warm by a block heater (Grant QBT2 Digital Block Heater, Gran Instruments, Cambridge, England) to prevent it from solidifying. 50 μL of agar solution was plated into each well of a 96-well plate (Nunc).

A soft agar top layer was prepared by mixing 1 part low melting point agarose in purified water (Sigma-Aldrich Inc.) with 3 parts of growth medium at 50°C in a water bath. The liquid agar was temporarily kept at 40°C. H1, H2 and H3 cells were trypsinised and quantified using a Countess Automated Cell Counter (Invitrogen, Waltham, MA, USA). A suspension of 8 x 10^4^ cells/mL in pre-warmed growth medium was prepared and mixed with equal parts of soft agar. The cell-containing soft agar was then transferred to a petri dish on a 37°C heat block and added on top of the base agar at 50 μL per well (2 x 10^3^ cells/well). The 96-well plates were kept in the fridge for 30 min before adding 100 μL growth medium containing CCT196969 in final concentrations of 0.01, 0.05, 0.1 and 1 μM for H1 and H2 and 0.05, 0.1, 0.5 and 1 μM for H3. After 10 days, microscopic images were captured with a Nikon TE2000 inverted microscope (Nikon Instruments Inc) using 10x and 20x objectives. Then, 20 μL of 0.1 mg/mL resazurin (Sigma-Aldrich Inc.) was added to each well and incubated for 4 h. The absorbance was measured at dual mode 560/590 nm using a scanning multi-well spectrophotometer (Victor 3 1420 multi-label counter, Perkin Elmer, Waltham, MA, USA), equipped with WorkOut 2.5 data analysis software. Each experiment was performed in triplicates (n = 6 per experiment per drug concentration). The results were analysed and IC_50_ doses were calculated using Prism 8 software (GraphPad Prism). Sphere diameters were measured in ImageJ software version 2.0.0 (National Institute of Health, Bethesda, MD; USA). Volumes were calculated in Excel (Microsoft) using the following formula: $\frac{4{\pi r}^{3}}{3}$.

### Cell migration assay

H1, H2 and H3 cells were seeded at a density of 3 x 10^4^ cells/well in Essen Bioscience ImageLock 96-well plates (Essen Bioscience Ltd., Hertfordshire, UK). After 48 h, a wound maker tool was employed to simultaneously create a consistent wound with a uniform width across the wells. All wells were washed with pre-heated growth medium before drug solutions were added to the wells in concentrations of 0.1, 0.5 and 1 μM. Imaging was performed every 2 h using the 10x objective in the IncuCyte Live Cell Imaging System (Essen BioScience Ltd.) for 72 h. Resulting images were analysed to find the wound width in μm using the IncuCyte Cell Migration Software Module (Essen BioScience Ltd.). The experiments were done in triplicates.

### Apoptosis assay by flow cytometry

Flow cytometry was performed to study drug effects on apoptosis. H1, H2 and H3 cell lines were plated in 6-well plates (Nunc) with a concentration of 3 x 10^5^ cells/well in 4 mL growth medium. After 24 h, CCT196969 was added to the wells in final concentrations of 1, 2 and 4 μM. Untreated cells were used as controls. 4 h before the collecting of cells, 4.08 μL of 30% H_2_O_2_, equivalent to a concentration of 10 μM, was added in one well to induce apoptosis and serve as an apoptotic control. After 72 h of incubation, growth medium was collected from the wells into tubes, and the cells were washed with phosphate buffered saline (PBS), trypsinated and added to the growth medium before centrifuging at 900 rpm for 5 min. After removal of the supernatant, cells from each sample were resuspended in 100 μL Annexin V binding buffer (Invitrogen), and 2 μL of Annexin V and propidium iodide (PI; AlexaFluor®488 Annexin v/dead cell apoptosis kit; Molecular Probes, Life Technologies, Waltham, MA, USA) were added to the samples. After 20 min incubation the samples were vortexed and analysed using a BD Accuri C6 flow cytometer (BD Bioscience, San Jose, CA, USA). Fluorescence in the FITC-A and PE-A channels were gated to a two-parameter histogram, and analysed using FloJo software (Tree Star Inc., Ashland, OR, USA). The experiment was repeated 3 times for each cell line.

### Western blot analysis

Western blots were performed to study change in protein levels in the MAPK, STAT3 and PI3K pathways after treatment with CCT196969. 1 x 10^6^ H1 and H3 cells were seeded into T25 culture flasks (Nunc). After 24 h cells were treated with 1, 2 and 4 μM CCT196969. Untreated cells were used as control. The cells were then incubated for 24 h, before collecting both floating and attached cells. The cells were lysed in 100 μL lysis buffer (radioimmunoprecipitation assay (RIPA) based buffer (Thermo Fischer Scientific) containing 10% PhosSTOP and CompleteMini protease inhibitors (Sigma-Aldrich Inc.)). Lysates were centrifuged at 13 000 rpm in 4°C for 5 min, and the resulting supernatants were used as final lysates. Protein levels were quantified using BCA protein assay (ThermoFischer Scientific) according to the manufacturer user guide. Lysates were electrophoresed on 10% or 15% SDS-polyacrylamide gel electrophoresis according to target protein sizes. Proteins were transferred to nitrocellulose membranes (GE Healthcare Life Science, Chicago, Illinois, USA), and then blocked in Tris-Buffered Saline (TBS), 0.1% Tween and 5% skim-milk for 1 h at room temperature. The membranes were incubated at 4°C overnight in antibody diluent (Thermo Fischer Scientific) mixed with the relevant antibody. The primary antibodies used were: p-ERK1/2 (Cell Signaling Technology, Inc, Danvers, MA, USA, cat. #4370, monoclonal, rabbit, dilution 1:1000, RRID:AB_2315112), p-MEK1/2 (Cell Signaling Technology, cat. #9154, monoclonal, rabbit, dilution 1:1000, RRID:AB_2138017), Stat3 (Cell Signaling Technology, cat. #4904, monoclonal, rabbit, dilution 1:2000, RRID:AB_331269), p-Stat3 (Cell Signaling Technology, cat. #9134, polyclonal, rabbit, dilution 1:1000, RRID:AB_331589), p-AKT (Cell Signaling Technology, cat. #4056, monoclonal, rabbit, dilution 1:1000, RRID:AB_331163), cleaved caspase-3 (Cell Signaling Technology, cat. #9664, monoclonal, rabbit, dilution 1:1000, RRID:AB_2070042) and loading control GAPDH (Abcam, Cambridge, UK, cat. #ab9485, polyclonal, rabbit, dilution 1:4000, RRID:AB_307275). The membranes were then washed in TBS-Tween 3 times before being incubated with a secondary antibody, Goat anti-Rabbit IgG (Invitrogen, cat. #31462, polyclonal, dilution 1:10 000, RRID:AB_228338), diluted in a blocking buffer. After 3 washing steps, proteins were detected using an enhanced chemiluminescence kit (Thermo Fischer Scientific) and a LAS3000 imaging system (FujiFilm, Saitama, Japan). Quantification of protein expression levels was based on band density measured in ImageJ software version 2.0.0 (National Institute of Health). The protein levels were normalised against the loading control and compared to the untreated controls. The experiments were performed in triplicates.

### Development of resistant cell lines and monolayer cell viability assay on resistant cell lines

To study the effect of CCT196969 on a BRAF inhibitor resistant cell line, H1 cells were seeded in increasing concentrations of vemurafenib until resistance was established. H1 cells of the equivalent passage were seeded in two separate T75 flasks (Nunc), one untreated as control, the other initially treated with 0.05 μM vemurafenib (Chemietek). The cells in both flasks were passaged 6 times in total, but in the latter flask the vemurafenib concentration was increased for each passage; 0.1, 0.25, 0.5, 1.0, 2.0 and 3.0 μM.

To verify the development of resistance towards vemurafenib, monolayer resazurin assays were performed for the H1 vemurafenib-naïve (H1) and H1 vemurafenib-resistant (H1-R) cell lines using increasing concentrations of vemurafenib. Thereafter, monolayer resazurin assays were performed on the H1 and H1-R cell lines of the same passage using 0.1, 0.5, 1 and 2 μM CCT196969. The experiments were performed in triplicates.

A Wm3248-DR (double resistant) BRAF-inhibitor resistant cell line was generated from the parental Wm3248 cell line by long-term culturing under continuous drug presence of the BRAF inhibitor encorafenib and the MEK inhibitor binimetinib, corresponding to approximately 10x IC_50_ concentration. Drug-naïve and drug-resistant cell lines were authenticated by STR profiling. After generation, resistant cell lines were maintained under continuous exposure to encorafenib and binimetinib. Monolayer resazurin assays, as previously described, were then performed on the cell lines using vemurafenib and CCT196969. The experiments were done in duplicates.

### Statistical analysis

Unpaired, 2-tailed t-tests were performed in Excel (Microsoft). A 2-tailed p<0.05 was considered statistically significant. Values presented in the figures represent means ± standard error of the mean (SEM) calculated in Prism 8 Software (GraphPad Software Inc).

## Results

### CCT196969 decreases viability of MBM cells in monolayer cultures

**Fig 1:**
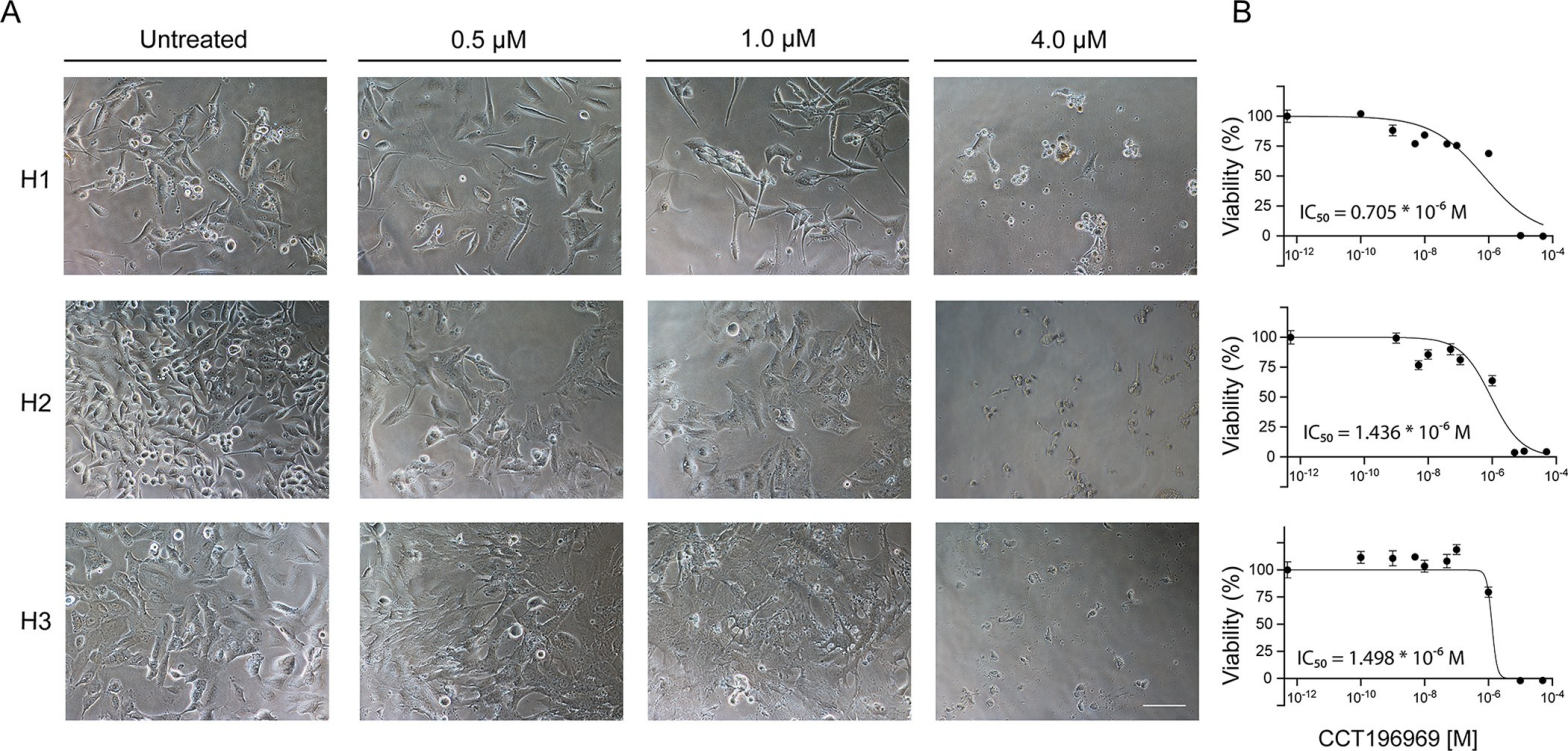
CCT196969 treatment reduces viability in MBM cell lines in a dose-dependent manner. (A) Representative microscopic images (20x objective) of H1, H2 and H3 cells grown in monolayers, exposed to increasing concentrations of CCT196969; untreated, 0.5, 1.0 and 4 μM, for 72 h. Scale bar = 100 μm. (B) Representative viability curves of the cell lines grown as monolayers, exposed to increasing concentrations of CCT196969. The mean IC_50_ dose value for CCT196969 was calculated from the experimental triplicate and is presented in the graph.

To explore whether CCT196969 affected cell growth in monolayer cultures, we performed cell viability assays. Changes in cell morphology after treatment were observed. In the 1 μM treatment group, H1 cells appeared elongated and more spindle-like, while H2 and H3 cells had a flatter, wider form and loss of structural integrity. Hardly any viable cells were present at 4 μM of CCT196969, and all cell lines displayed cell shrinkage and intracellular fragmentation (Fig 1A).

Treatment with CCT196969 reduced viability in a dose-dependent manner in the H1, H2, H3, H6, H10 and Wm3248 cell lines (Fig 1B, S1 Fig). The IC_50_ doses were calculated to be 0.7, 1.4, 1.5, 2.6, 1.2 and 0.18 μM, respectively. At a concentration of 10–50 μM, no viable cells were detected in any cell line (Fig 1B, S1 Fig).

### CCT196969 decreases viability of MBM cells grown as tumour spheres

**Fig 2:**
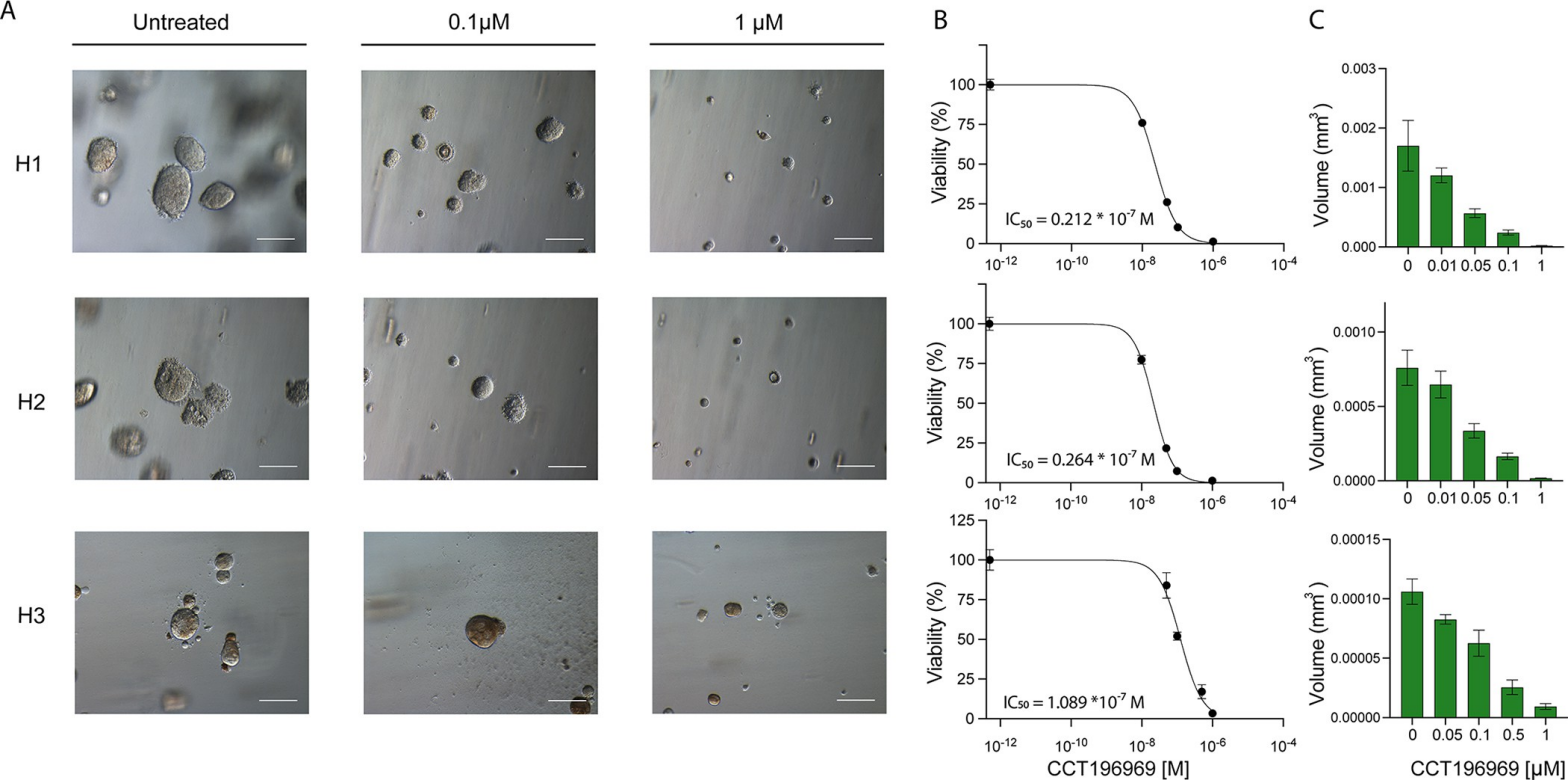
CCT196969 treatment reduces tumour sphere growth in MBM cell lines in a dose-dependent manner. (A) Representative phase-contrast microscopic images (20x objective) of the H1, H2 and H3 cell lines at different concentrations of CCT196969; untreated, 0.1 and 1 μM. Scale bars = 100 μm. (B) Representative viability curves of the 3 cell lines with the mean IC_50_ dose of the triplicates. (C) Mean tumour sphere volumes (in mm^3^) measured after 10 days exposure to different CCT196969 concentrations: untreated, 0.01, 0.05, 0.1 and 1 μM for the H1 and H2 cell lines, and untreated, 0.05, 0.1, 0.5 and 1 for the H3 cell line.

To examine the effects of CCT196969 on colony formation and anchorage-independent growth, we conducted a tumour sphere assay. After 10 days of culturing in agar, the untreated cells formed spheres ranging from 100–200 μm in diameter (Fig 2A). The sphere sizes in treated groups were negatively correlated with increasing doses when compared to untreated spheres (Fig 2A and 2C). The calculated IC_50_ values for the H1, H2 and H3 cell lines were 0.02, 0.03 and 0.1 μM, respectively, showing that tumour spheres were more drug sensitive than the monolayer cultures (Fig 2B).

### CCT196969 decreases MBM cell migration

**Fig 3:**
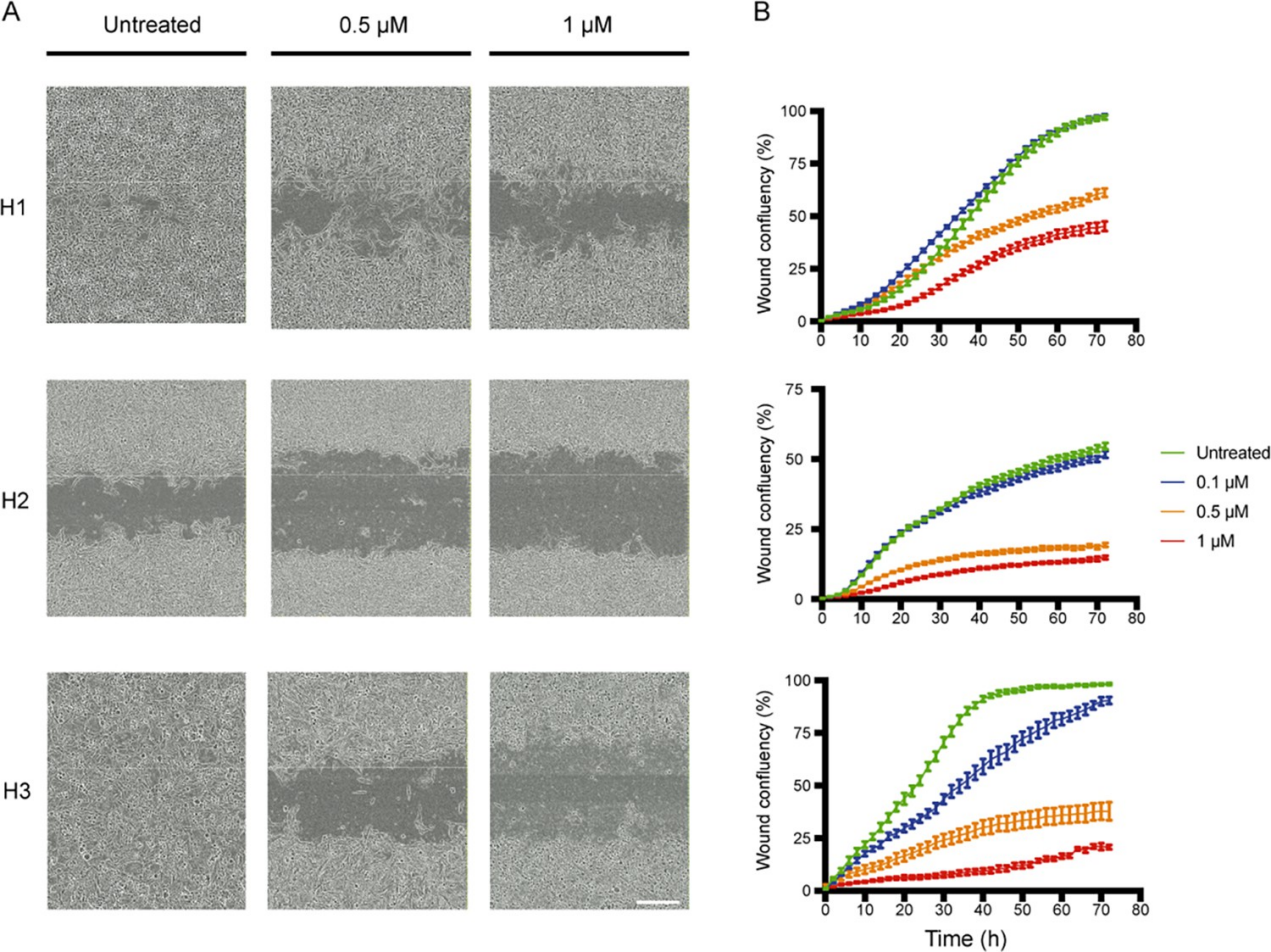
CCT196969 reduces migration in MBM cell lines. (A) Representative microscopic images (10x objective) of the H1, H2 and H3 cell lines displaying differences in wound confluency at 72 h, untreated or exposed to 0.5 or 1 μM of CCT196969. Scale bar = 300 μm. (B) Representative graphs of the wound confluency in the H1, H2 and H3 cell lines during 72 h. The experiments were done in triplicates.

We performed a scratch wound assay to demonstrate drug effects on cell migration. CCT196969 treatment decreased the migratory abilities of the H1, H2 and H3 cell lines in a dose-dependent manner (Fig 3). The wounds closed completely within 72 h in the untreated H1 and H3 cell lines. Wound confluency after 72 h was between 20–40% and 20% in 1 μM-treated H1 and H3 cells, respectively. The untreated H2 cells had a wound confluency of 65% after 72 h, whereas cells treated with 1 μM had confluency of approximately 20%.

### CCT196969 induces apoptosis in MBM cells

**Fig 4:**
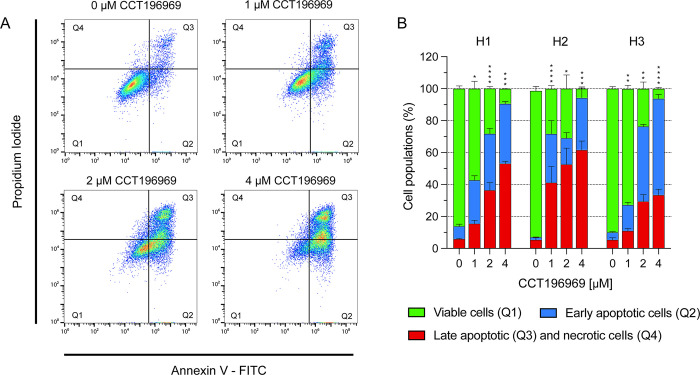
CCT196969 induces apoptosis in MBM cell lines. Annexin V labels apoptotic cells and propidium iodide labels necrotic cells. (A) Representative dot plots of the H1 cell line treated with selected concentrations of CCT196969; untreated, 1, 2 and 4 μM. (B) Percentage of viable cells, early apoptotic and late apoptotic and dead cells in the H1, H2 and H3 cell lines. The experiments were done in triplicates. Abbreviations: Q1: Viable cells, Q2: Early apoptotic cells, Q3: Late apoptotic cells, Q4: Necrotic cells. *: p < 0.05, **: p < 0.01, ***: p < 0.001, ****: p < 0.0001.

**Fig 5:**
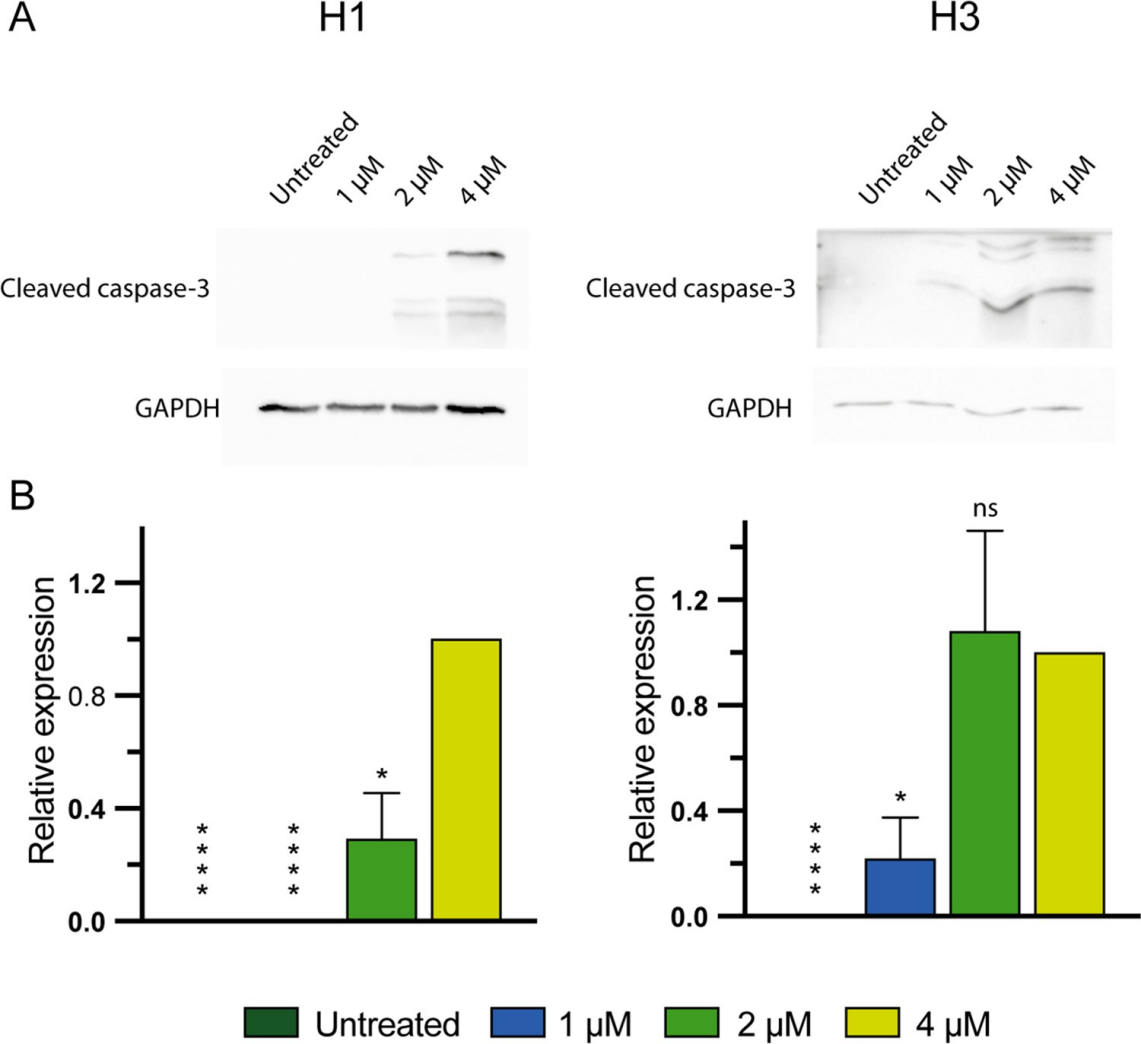
CCT196969 upregulates expression of cleaved caspase-3 in MBM cell lines. (A) Western blots showing cleaved caspase-3 from H1 and H3 cells, untreated or treated with 1, 2 or 4 μM CCT196969 for 24 h. (B) Quantification of Western blots against the loading control GAPDH in ratio with the bands of the highest concentration of CCT196969. Due to no expression of cleaved caspase-3 in the untreated groups, expression of cleaved caspase-3 in the highest concentration group was set to 1.0. The experiments were done in triplicates. Abbreviations: ns: not significant, *: p < 0.05, ****: p < 0.0001.

To assess drug effects on apoptosis and cell survival, we performed an apoptosis assay by flow cytometry. CCT196969 induced apoptosis in the H1, H2 and H3 cell lines (Fig 4A, S2 Fig). 4 μM CCT196969 induced early and late apoptosis in about 90%, 94%, and 94% of each of the cell lines, respectively, compared to 14%, 7%, and 10% apoptotic cells in untreated controls. For untreated cells, between 86–92% of the cells were viable in the cell lines, compared to 5–10% after treatment with 4 μM CCT196969. The increase in apoptosis after treatment was significant, compared to untreated controls (Fig 4B). Flow cytometry results were validated by Western blots showing an increase in cleaved caspase-3 after CCT196969 treatment of H1 and H3 cells with CCT196969 (Fig 5). Cleaved caspase-3 was not detected in the untreated groups.

### CCT196969 reduces phosphorylation of key kinases in the MAPK, STAT3 and PI3K signalling pathways

**Fig 6:**
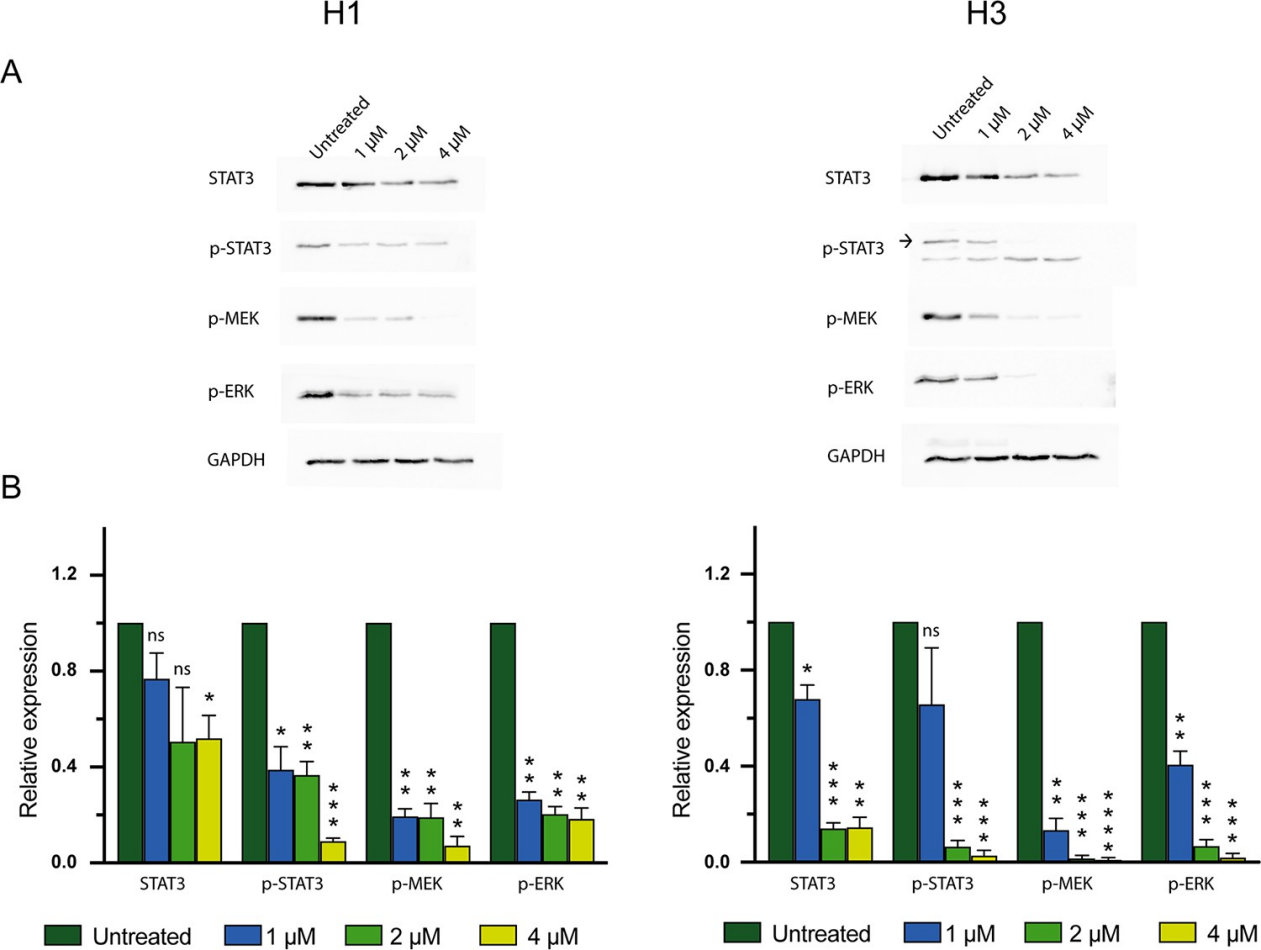
CCT196969 downregulates expression of STAT3, p-STAT3, p-MEK and p-ERK in MBM cell lines. (A) Western blots from H1 and H3 cells, untreated or treated with selected doses of CCT196969; 1, 2 and 4 μM. (B) Quantification of Western blots normalised against the loading control GAPDH and in ratio with the untreated control. The experiments were done in triplicates. The graphs show the mean with SEM. Abbreviations: ns: not significant, *: p < 0.05, **: p < 0.01, ***: p < 0.001, ****: p < 0.0001.

To investigate the effects on MAPK, STAT3 and PI3K signalling pathways upon treatment with CCT196969, H1 and H3 cells were treated with CCT196969 for 24 h and analysed by Western blotting. We observed a significant decrease in expression of STAT3, p-STAT3, p-MEK and p-ERK in both cell lines upon treatment with CCT196969 (Fig 6). In addition, p-AKT protein level was downregulated in the H1 cell line (S3 Fig).

### CCT196969 reduces viability of BRAF inhibitor resistant cells

**Fig 7:**
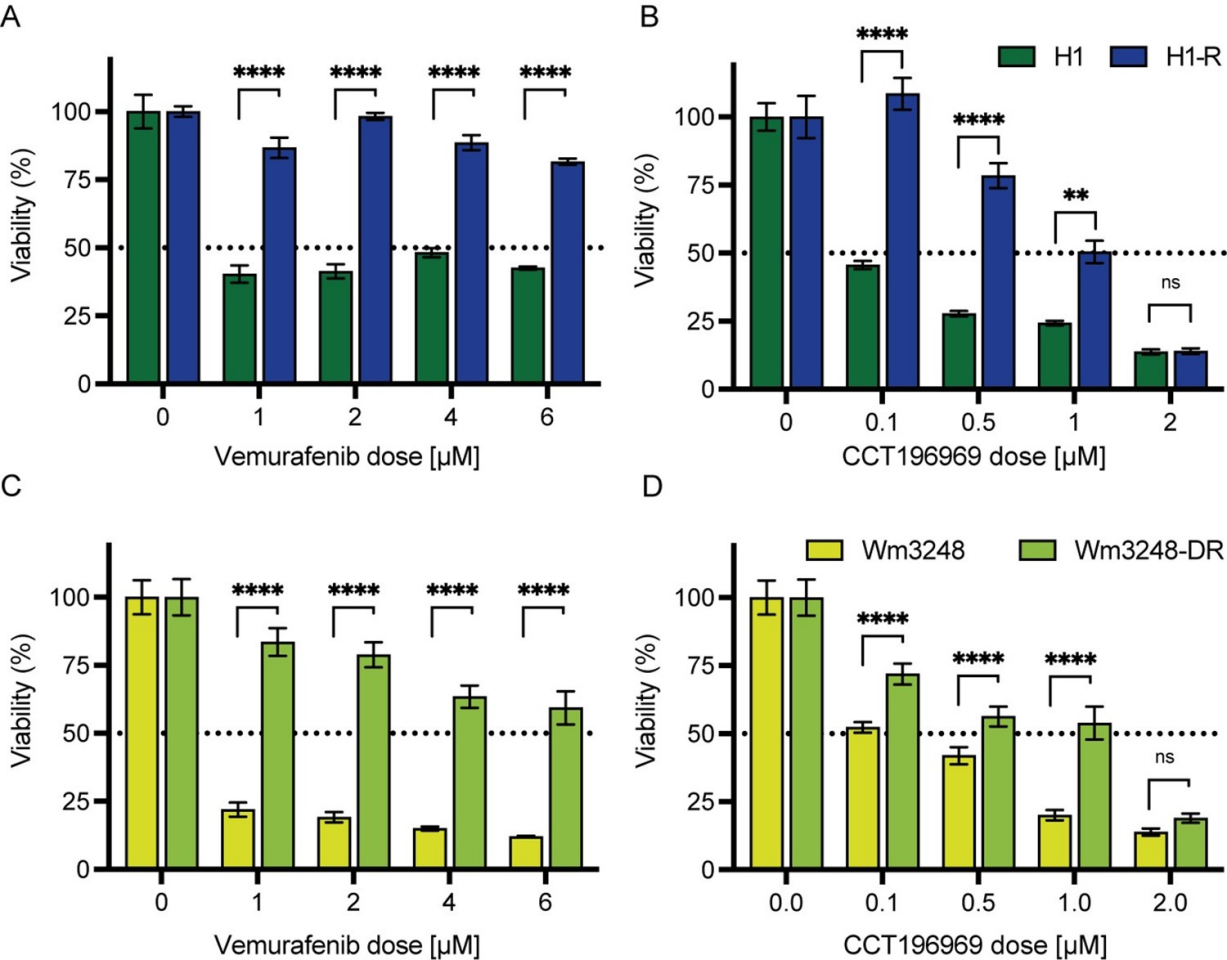
Cell viability in the vemurafenib-naïve and resistant cell lines after exposure to increasing concentrations of vemurafenib (A, C) and CCT196969 (B, D). (A) Dose response curves after exposure to vemurafenib for H1 and H1-R using the following concentrations: untreated, 1, 2, 4 and 6 μM. (B) Dose response curves for H1 and H1-R after exposure to CCT196969 using the following concentrations: untreated, 0.1, 0.5, 1 and 2 μM. (C) Dose response curves after exposure to vemurafenib for Wm3248 and Wm3248-DR using the following concentrations: untreated, 1, 2, 4 and 6 μM. (D) Dose response curves for Wm3248 and Wm3248-DR after exposure to CCT196969 using the following concentrations: untreated, 0.1, 0.5, 1 and 2 μM. Abbreviations: ns: not significant, **: p < 0.01, ****: p < 0.0001.

To assess the effects of CCT196969 on BRAF inhibitor resistant cell lines, we first developed BRAF inhibitor resistance in the H1 and Wm3248 cell lines. Viability studies confirmed significant differences in vemurafenib sensitivity between the H1 and H1-R cell lines, and for the Wm3248 and Wm3248-DR cell lines (Fig 7A and 7C).

To the investigate the effects of CCT196969 on vemurafenib-naïve metastatic melanoma cell lines and their drug-resistant counterparts, viability studies were performed. For the lowest concentrations of CCT196969, significant differences in viability were observed for the H1 and H1-R cell lines, similar to what was also found for Wm3248 and Wm3248-DR cell lines (S1 Table). Both the vemurafenib-naïve and the resistant cell lines showed a dose-dependent reduction of viability when treated with CCT196969 (Fig 7B and 7D), although the effect on the resistant cell lines were less compared to the vemurafenib-naïve cells when treated with 0.1, 0.5 and 1 μM CCT196969. There were no significant differences in viability between the sensitive and resistant cell lines when treated with 2 μM CCT196969 (Fig 7B and 7D).

## Discussion

Around 90% of melanomas express abnormal activation of the MAPK pathway [8], providing a strong rationale for clinical use of BRAF and MEK inhibitors. However, primary and secondary resistance mechanisms significantly impede the clinical utility of these drugs, and second-line treatments are scarce. Developing new therapies, one should also take into account that MBMs have distinct expression patterns as compared to extracranial metastases [27]. PI3K and STAT3 pathway overexpression drive development of MBM and also confers survival and resistance during MAPK treatment [28, 29]. Here, we show for the first time that CCT196969 targets multiple key signalling pathways in MBM and effectively inhibits growth, migration, and induces apoptosis in several BRAF inhibitor sensitive and resistant MBM cell lines *in vitro*.

Viability studies demonstrated that the IC_50_ doses were in the range of 0.18–2.6 μM, which is in line with previous studies using CCT196969 and other SFK inhibitors in primary melanoma cell lines [15, 24, 30] and metastatic melanoma [31]. Interestingly, the H3 cell line, which harbours a *BRAF*^*L577F*^ mutation of unknown clinical relevance and mutations in *NRAS* and *EGFR*, displayed an IC_50_ dose similar to the *BRAF*^*V600E*^ mutated cell lines. It has previously been shown that the IC_50_ dose of the BRAF inhibitor vemurafenib was around 10-fold higher in the H3 cell line compared to the H1 cells [32]. This suggests that CCT196969 may be an effective treatment in melanomas that carry other pathogenic mutations than *BRAF*^*V600E*^. In addition, treatment of *NRAS*-mutated cell lines with BRAF inhibitors is known to reactivate the MAPK pathway through BRAF/CRAF dimerisation [12, 13], thereby inducing increased signalling and proliferation. This reactivation may be avoided by simultaneously targeting SFK and several RAFs using CCT196969.

Tumour spheres are generally more representative than monolayers in mimicking tumour growth *in vivo* [33]. CCT196969 treatment of tumour spheres inhibited growth with IC_50_ doses around 15–47 times lower as compared to monolayers. This is in line with other findings [34] and indicates that the MBM cells are more drug sensitive when grown in an anchorage independent manner.

Melanoma is well known for its unique metastatic properties, exhibiting increased migration and invasiveness, as well as aggressive local invasion and early metastatic dissemination [35]. It has been shown that MBMs may be found in more than 70% of autopsies in patients with advanced melanoma [36]. At doses less than the IC_50_ doses, we found that CCT196969 inhibited MBM cell migration in H1, H2 and H3 cells. This indicates that CCT196969 at lower concentrations inhibits migration but may also be partly due to reduced cell proliferation.

Previous reports have also suggested that SFK inhibitors decreased migration and invasion in melanoma cells [37, 38]. The Src/Abl inhibitor dasatinib eliminated all SFK activity in primary melanoma, with no concurrent effects on ERK1/2 protein kinases. Cell migration and invasion were inhibited before affecting cell viability or cell proliferation [39]. Inhibition of cell migration by SFKs and effects on melanoma cell proliferation have also been demonstrated [15, 30], yet the literature remains inconclusive as to possible *in vivo* effects [40]. Taken together, SFK activity seems to inhibit cell migration in MBM cells and in primary melanoma, at doses below IC_50_ and without affecting cell proliferation.

CCT196969 effectively induced apoptosis in the H1, H2 and H3 cell lines in a dose dependent manner, with 90% to 95% of the cells being apoptotic at a concentration of 4 μM. Morphological examinations demonstrated typical apoptotic traits. Morphologically, the H1 cell line displayed an elongated, spindle-like shape after treatment, while the H2 and H3 cells appeared wider with reduced focal adhesion. Changes observed in the H1 cells resemble an epithelial-mesenchymal transition (EMT), which has been previously reported for MBM cells treated with MAPK inhibitors [26, 41].

Apoptosis was confirmed by Western blotting, showing increased levels of the apoptotic marker cleaved caspase-3 in cells treated with 2 μM and 4 μM CCT196969. Cleavage of caspase-3 leads to activation of the caspase cascade, which plays a crucial role in both the intrinsic and extrinsic apoptotic pathways, and thus execution of apoptosis [42]. Previous work has shown increase in cleaved caspase-3 in vemurafenib-resistant melanoma cell lines after treatment with 1 μM CCT196969 [24].

CCT196969 is a pan-RAF and SFK inhibitor, targeting both the MAPK and STAT3 pathways. CCT196969 has been shown to target mutated BRAF, CRAF and SFKs in *BRAF*-mutated primary melanoma cell lines, resulting in decreased expression levels of p-MEK and p-ERK [24]. In our study, CCT196969 downregulated p-ERK, p-MEK, p-STAT3 and STAT3 in the H1 and H3 cell lines. The H3 cell line has mutations in *NRAS* and *EGFR*, making both the MAPK and STAT3 pathways relevant targets. Targeting of the STAT3 pathway is a crucial point, as activation leads to increased production of vascular endothelial growth factor (VEGF), an angiogenic factor important for tumour development, invasion, and metastasis [15, 43]. Both pathways have also been shown to be upregulated in BRAF inhibitor resistant cells [15, 44]. Due to STAT3 and MAPK pathway crosstalk, inhibition of one pathway may lead to upregulation of the other; targeting both pathways simultaneously would likely exhibit a stronger inhibitory effect on tumours [45].

Our results also indicate that CCT196969 may have an inhibitory effect in the PI3K pathway in MBM through downregulation of p-AKT (S3 Fig). Hyperactivation of the PI3K pathway is more common in intracranial than extracranial metastases [46], and is therefore a key target in MBM patients. Whether CCT196969 would be effective against tumours gaining resistance through PI3K-signalling remains unknown. As CCT196969 has been suggested as a potential second line treatment for BRAF inhibitor resistant melanoma and first line therapy for *NRAS-* and *BRAF*-mutated melanoma [24], combination therapy with PI3K inhibitors should be investigated in the future.

CCT196969 inhibited cell viability in a dose-dependent manner in our vemurafenib resistant metastatic cell lines. There were no significant differences in cell viability between the BRAF inhibitor resistant and sensitive cell lines at 2 μM, indicating that CCT196969 could be effective against BRAF inhibitor resistant melanoma. We did not investigate the mechanisms responsible for the acquired resistance, but upregulation of the MAPK, STAT3 or PI3K pathways all serve as likely mechanisms. A clinical study using patient-derived xenografts from patients obtained before and after receiving vemurafenib treatment, found elevated levels of p-SFK and p-ERK in the vemurafenib resistant tumours after treatment [24]. The resistant tumours were not sensitive to the vemurafenib analogue PLX4720 but were still responsive to treatment with CCT196969 [24].

In a previous study, EGFR was hyperphosphorylated in 4 out of 5 tumours from patients with acquired or intrinsic resistance to vemurafenib, leading to a hyperactivation of the STAT3 pathway [15]. In the present study, CCT196969 displayed anti-proliferative and apoptotic effects in the H3 cell line harbouring an *EGFR* pathogenic mutation, and treatment with CCT196969 downregulated p-STAT3. CCT196969 seems to be an effective inhibitor of cell viability in *EGFR*-mutated melanoma, as well as in BRAF inhibitor resistant melanoma which has achieved resistance with upregulation of the STAT3 pathway.

Drug penetrance through the blood brain barrier (BBB) is a central challenge in treatment of intracranial metastases. Previous studies on CCT196969 have demonstrated conflicting results on BBB penetrability [31]. *In vitro* experiments showed that CCT196969 was not a substrate for two important efflux transporters (P-glycoprotein (P-gp) and breast cancer resistance protein (Bcrp)), but in vivo studies found enhanced brain distribution without these transporters [31]. To establish the potential clinical value of CCT196969, toxicity issues, drug dosage, penetrance of the BBB and the significance of drug efflux pumps in drug delivery should be investigated before further *in vivo* treatment experiments are carried out.

In conclusion, we found CCT196969 to be an effective inhibitor of MBM proliferation, growth, and survival *in vitro*. Further *in vivo* studies should be performed to determine its therapeutic potential, as it has been shown to have good bioavailability and to be well tolerated in mice [24]. CCT196969 may serve as a potential first line treatment for patients with *NRAS*- or *EGFR*-mutated MBMs, and as second line treatment for patients with intrinsic or acquired resistance towards MAPK inhibitors.

## Supporting information

S1 Fig Viability curves of cells grown as monolayers after treatment with increasing doses of CCT196969. (A) Representative graph of the H6 cell line treated with increasing doses of CCT196969 (0.0001–50 μM) for 72 h. (B) Representative graph of the H10 cell line treated with increasing doses of CCT196969 (0.0001–50 μM) for 72 h. The experiments were performed in triplicates. (C) Representative graph of the Wm3248 cell line treated with increasing doses of CCT196969 (0.0001–50 μM) for 72 h. The experiments were performed in duplicates.
(TIF)

S2 Fig Flow cytometric analysis of apoptosis in the H2 and H3 MBM cell lines after treatment with CCT196969. Annexin V marks apoptotic cells and Propidium Iodide marks necrotic cells. (A) Dot plots of the H2 cell line treated with selected concentrations of CCT196969; untreated, 1, 2 and 4 μM. (B) Dot plots of the H3 cell line treated with selected concentrations of CCT196969; untreated, 1, 2 and 4 μM. The experiments were done in triplicates. Abbreviations: Q1: Viable cells, Q2: Early apoptotic cells, Q3: Late apoptotic cells, Q4: Necrotic cells.
(TIF)

S3 Fig CCT196969 downregulates expression of p-AKT in the H1 cell line. (A) Western blot bands from H1 cells treated with selected doses CCT196969; untreated, 1, 2 and 4 μM. (B) Quantification of protein bands normalised against the loading control GAPDH and in ratio with the untreated control. The experiments were done in triplicates. Abbreviations: *: p < 0.05, ***: p < 0.001.
(TIF)

S1 Table Statistical testing of the effects of single drug treatment on cell lines. Statistical testing of cell survival, comparing untreated cells with cells treated with either CCT196969 or vemurafenib at different concentrations. Results show p-values from T-test comparisons of cells treated with the given concentrations vs untreated control of corresponding cell line.
(TIF)

S1 Raw images (TIF)
