# Supplementary figures and images for: CCT196969 effectively inhibits growth and survival of melanoma brain metastasis cells
Source: PLoS One. 2022 Sep 9;17(9):e0273711. doi: 10.1371/journal.pone.0273711 (PMC9462752; doi:10.1371/journal.pone.0273711)

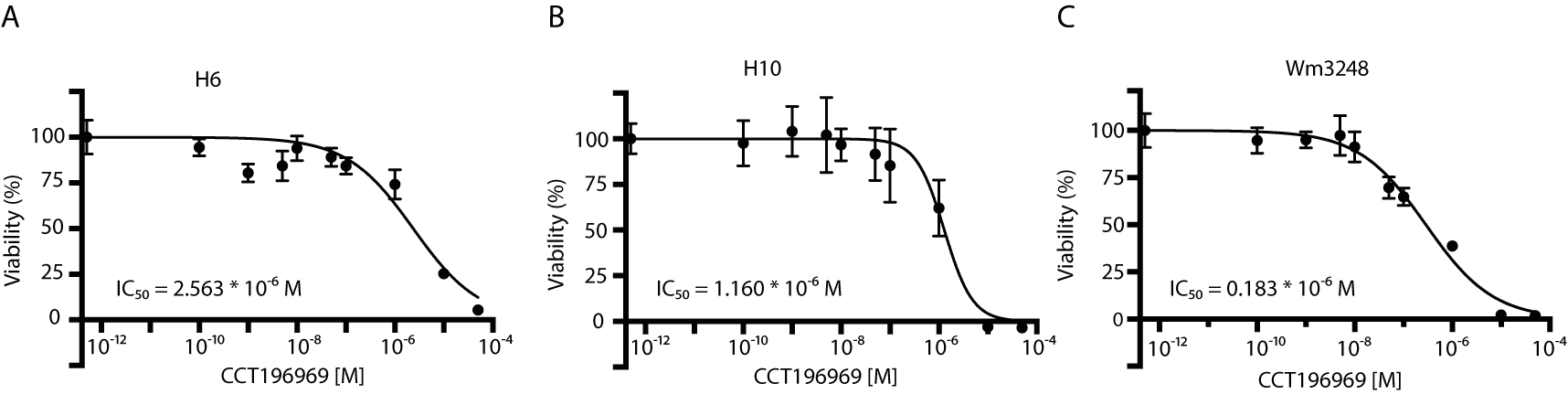

Supplement: S1 Fig — (A) Representative graph of the H6 cell line treated with increasing doses of CCT196969 (0.0001–50 μM) for 72 h. (B) Representative graph of the H10 cell line treated with increasing doses of CCT196969 (0.0001–50 μM) for 72 h. The experiments were performed in triplicates. (C) Representative graph of the Wm3248 cell line treated with increasing doses of CCT196969 (0.0001–50 μM) for 72 h. The experiments were performed in duplicates. (TIF) [file pone.0273711.s001.tif]

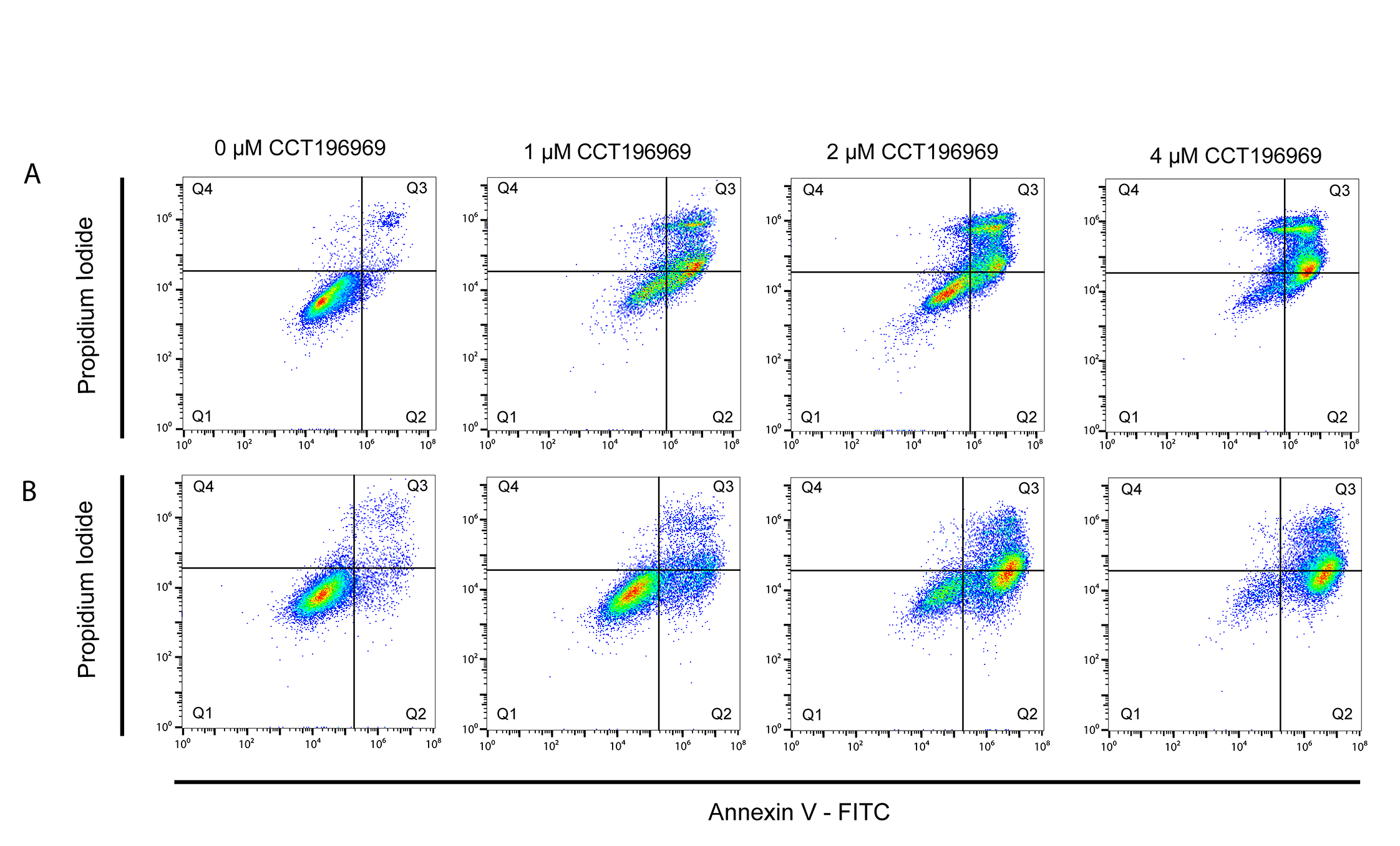

Supplement: S2 Fig — Annexin V marks apoptotic cells and Propidium Iodide marks necrotic cells. (A) Dot plots of the H2 cell line treated with selected concentrations of CCT196969; untreated, 1, 2 and 4 μM. (B) Dot plots of the H3 cell line treated with selected concentrations of CCT196969; untreated, 1, 2 and 4 μM. The experiments were done in triplicates. Abbreviations: Q1: Viable cells, Q2: Early apoptotic cells, Q3: Late apoptotic cells, Q4: Necrotic cells. (TIF) [file pone.0273711.s002.tif]

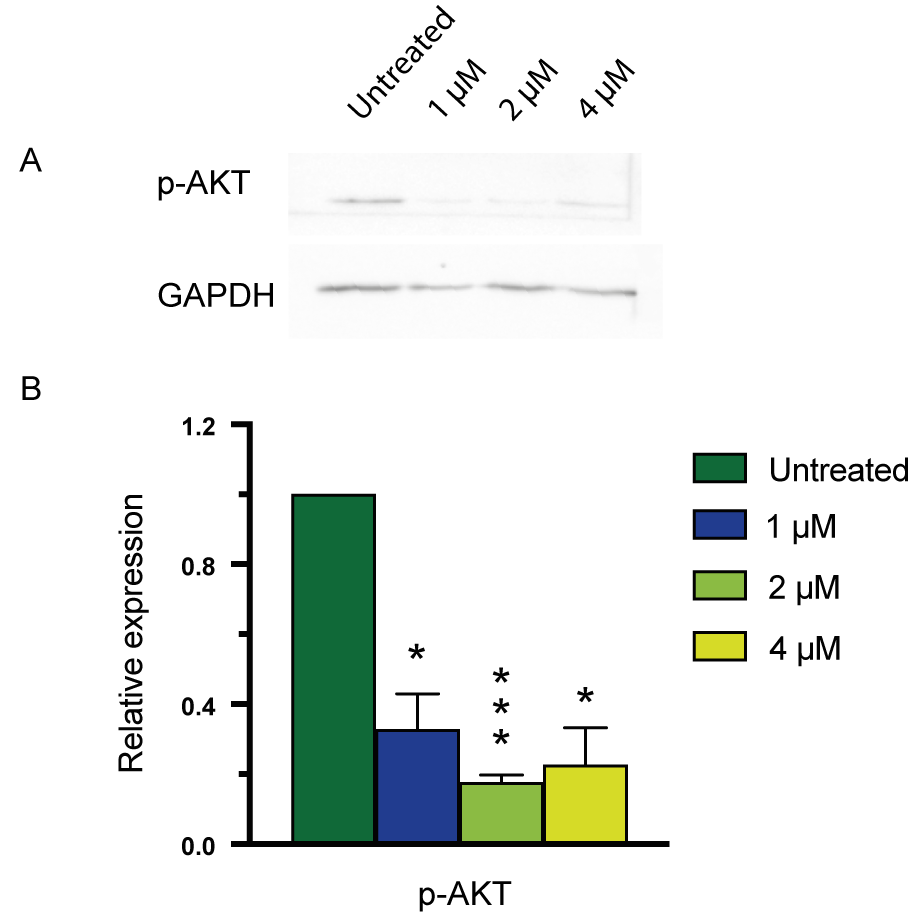

Supplement: S3 Fig — (A) Western blot bands from H1 cells treated with selected doses CCT196969; untreated, 1, 2 and 4 μM. (B) Quantification of protein bands normalised against the loading control GAPDH and in ratio with the untreated control. The experiments were done in triplicates. Abbreviations: *: p < 0.05, ***: p < 0.001. (TIF) [file pone.0273711.s003.tif]

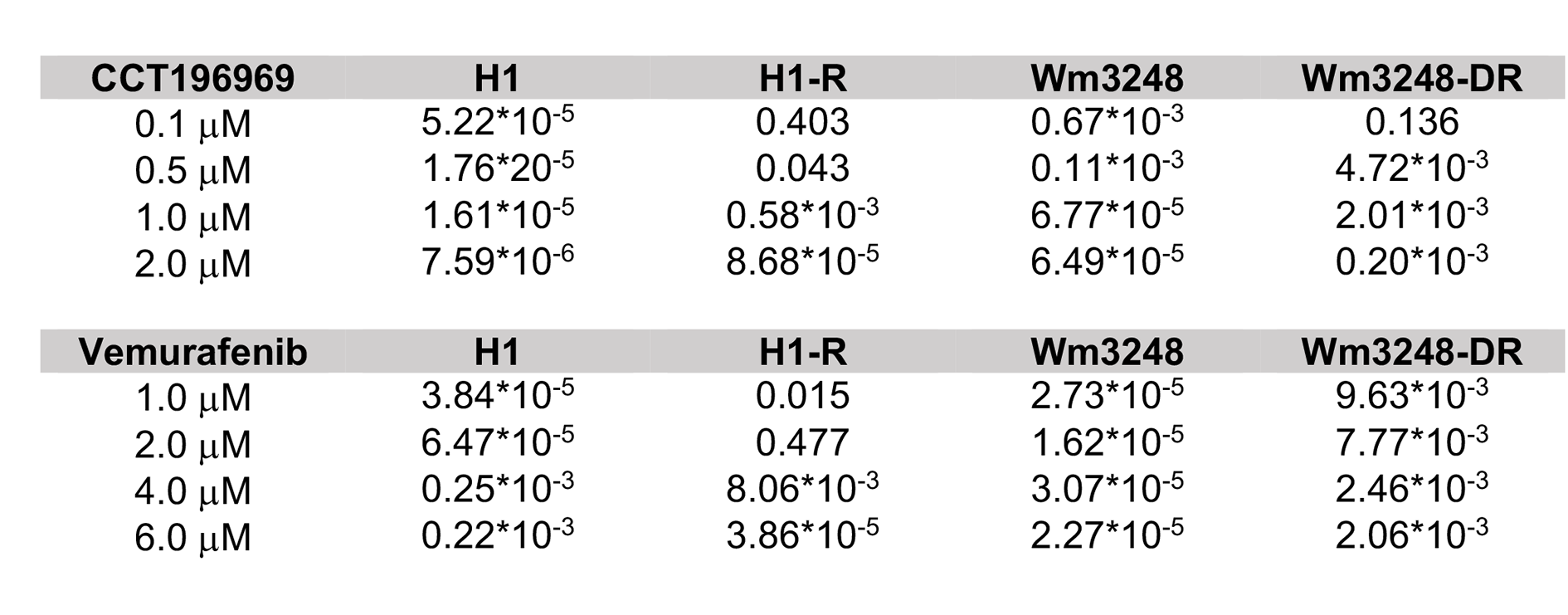

Supplement: S1 Table — Statistical testing of cell survival, comparing untreated cells with cells treated with either CCT196969 or vemurafenib at different concentrations. Results show p-values from T-test comparisons of cells treated with the given concentrations vs untreated control of corresponding cell line. (TIF) [file pone.0273711.s004.tif]

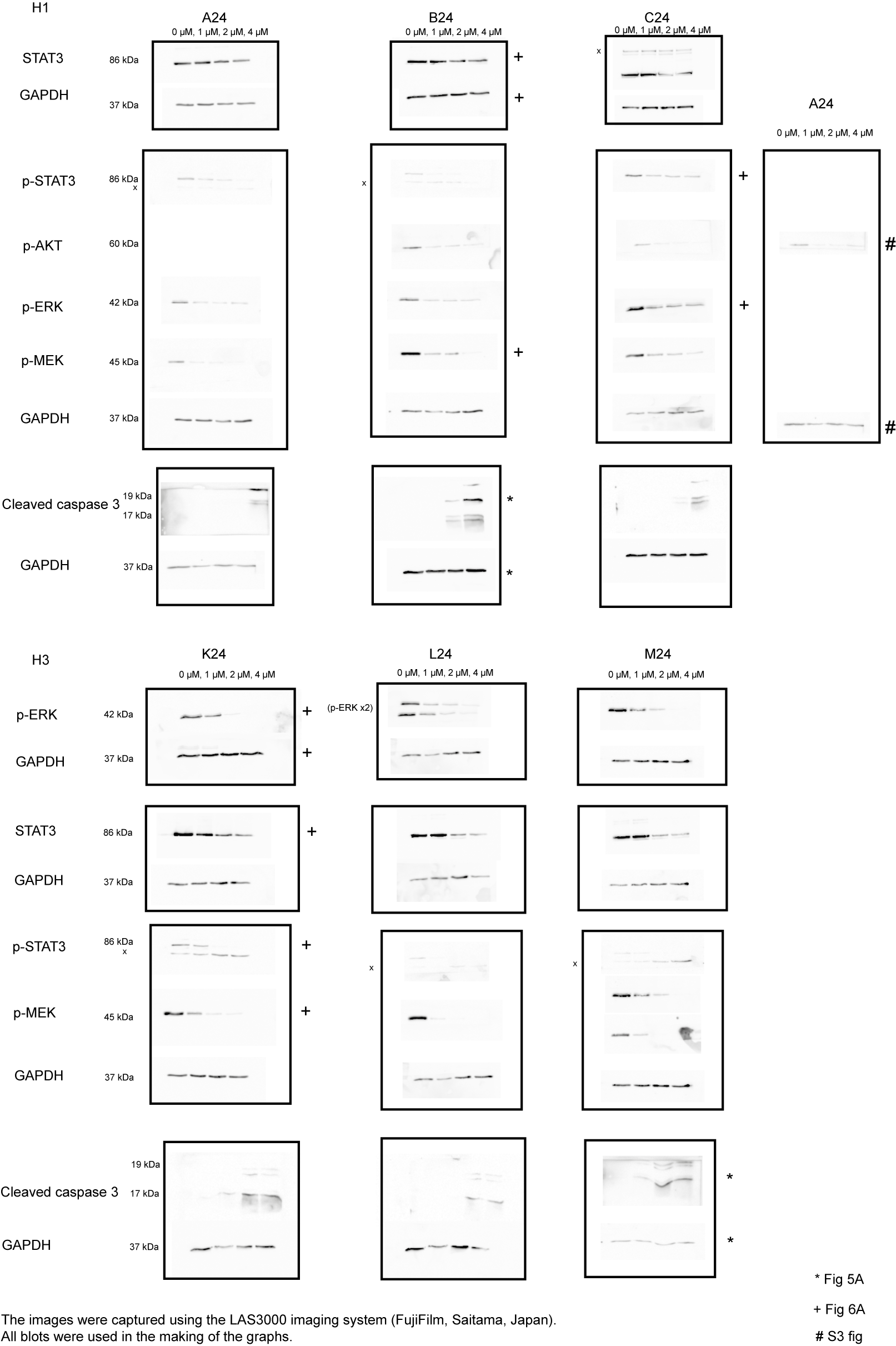

Supplement: S1 Raw images — (TIF) [file pone.0273711.s005.tif]
